# The Medical News and the New York Medical Journal

**Published:** 1905-11

**Authors:** 


### The Medical News and the New York Medical Journal.

THE *Medical News* makes editorial announcement in its issue of November 11 1905, that with the beginning of next year it will pass from the hands of Lea, Brothers and Company, into those of the A. R. Elliott Publishing Company. The latter will merge the *News* with the *New York Medical Journal,* and so, after sixty-two years since founding the *News* the proprietorship of the house of Lea retires from its publication.

The prospective merger is reaffirmed in the editorial columns of the *New York Medical Journal* of even date with the notice in the *News,* with the statement that the consolidation is made purely in the interest of medical journalism, and with the editorial assurances that "all the advantages that accrued from the acquisition of the *Philadelphia Medical Journal* may confidently be counted on to be amplified as the result of our incorporating the *Medical News* with the present consolidated Journal."

This may be taken to mean that Dr. Frank P. Foster, the distinguished editor of the *New York Medical Journal,* likewise dean of the medical editorial profession of America, will continue as editor-in-chief of the consolidated journalistic triad,—which of course means also success to the enterprise. We are sorry, we must confess, to part with the *News;* its honorable career is a credit to American medical journalism; but since it is decreed to go, it could scarcely have chosen a more graceful or dignified method of exit.

